# **Patents on hospital medical and dental equipment (EMHO). Question and answer tool** ^**1**^

**DOI:** 10.1590/s0102-865020190010000008

**Published:** 2019-02-14

**Authors:** Elisiane Kiel Lee, Lydia Masako Ferreira, Elaine Kawano Horibe

**Affiliations:** I Fellow Master degree, Postgraduate Program in Technology and Management of Tissue Regeneration, Medical School, Universidade Federal de São Paulo (UNIFESP), Brazil. Conception and design of the study; Acquisition, analysis and interpretation of data; technical procedures; statistics analysis; manuscript preparation and writing.; II Head, Full Professor, Division of Plastic Surgery, UNIFESP, Researcher 1A-CNPq, Director Medicine III-CAPES, Sao Paulo-SP, Brazil. Conception and design of the study, critical revision, final approval.; III PhD, Postgraduate Program in Technology and Management of Tissue Regeneration, Medical School, UNIFESP, Sao Paulo-SP, Brazil. Analysis and interpretation of data, statistics analysis, critical revision, final approval.

**Keywords:** Hospitals, Medicine, Equipment and Supplies.

## Abstract

**Purpose:**

To create a question and answer tool on patents on EMHO.

**Methods:**

Was used the Thinking Design methodology divided into four phases: Discovery, Definition, Development and Delivery. Discovery Phase: Desk research was carried out in: SciELO, Pubmed, LILACS, Google and Google Scholar. Once the target audience was selected, the interviews were conducted. Definition Phase: the interviewees’ difficulties were mapped, on an Excel spreadsheet. Development Phase: a brainstorming was conducted with the public interviewed. Delivery Phase: the prototype, validation and final elaboration of the tool were made.

**Results:**

Discovery Phase: 10 inventors were identified and the interviews were carried out. Definition Phase: 80% of the interviewees determined lack of information as one of the problems. The main content was defined as: the patent process, from the beginning of the idea to the deposit (70%), search for precedence (40%) and informing partners (30%). Development Phase: with the brainstorming, the tool type was defined as an interactive site. Delivery Phase: a prototype with content framework and an interactive video was presented for validation. After approval, the interactive website was developed, which was made available to the public.

**Conclusion:**

A question and answer tool on patents in EMHO was developed.

## Introduction

 Intellectual Property (IP) is the area of law that seeks to protect the creations of the human mind. It is a legal construction that comes from the right of ownership, to give exclusivity of use to the market of the intellectually-created assets^1^. IP is divided into three parts: Copyright, Industrial Property and *Sui Generis* Protection^2^.

With regard to industrial property, we can mention patents, which are documents that aim at protecting an invention for a determined period of time^3^. According to the World Intellectual Property Organization^4^ the patent is a right granted to an invention, which usually provides a new way of doing something or a new technical solution to a problem. In order to obtain a patent, technical information on the invention is required and must be made publicly available under a specific request.

A country’s innovative capacity is measured by number of patents^5^. They make up the legal system to ensure innovation, and it is crucial for manufacturers, as they allow them to recover the high initial costs of their research and development (R&D), by ensuring monopoly over creation^6^.

WIPO^4^, in its statistical base, released the ranking of the 10 countries considered the largest patent applicants, with Brazil in the tenth position, with 30.884 processes and China ranking first place, with 825.136 patents. When considering areas of technology, such as: computing, optics, and medical technology, the United States rank first place in the medical field. With regard to hospital medical and dental (EMHO) equipment, the United States take 40% of the economic market. Brazil is in 11^th^ place, with 1.4% of the market^7^.

EMHO is estimated in about US$ 210 billion, concentrated in developed countries, and represent great interest for the development of relevant technology for the Health area^7^. The economic and social importance of innovation in medical equipment is unequivocal and requires, besides intellectual protection, specific regularization to be placed in the market^8^.

In the words of Schwertner^9^ and Jorge de Paula Costa Ávila, the former president of the National Institute of Industrial Property (INPI), one of the problems faced by the development of innovation and patent filing is the lack of information on intellectual property^10^.

Pinheiro-Machado and Freitas^11^ stated that INPI, in the last 20 years, since the passing of the industrial property law 1996, is investing in education, mainly after the innovation law, in 2004. A survey carried out by INPI, in 2015, found that 60% of the largest applicants of invention patents, coming from the research activities carried out in the country, are education and research institutions.

There are few websites focusing on patents on EMHO. The WIPO and European Patent Office (EPO) bring some regulations on medical devices, emphasizing that Brazilian legislation will always be in force, so these regulations should be studied from a Brazilian point of view. INPI has patent literature in general. There are scientific papers, in patents in EMHO, analyzed internationally. In Brazil, the papers are only focused on patents or on EMHO, but none relate these two topics.

Given the needs described above, this work aimed at developing a question and answer tool that makes access to information on filing and obtaining patents easy, with an emphasis on EMHO, aimed at EMHO researchers and inventors.

## Methods

 This study is prospective and was developed in the Department of Science, Technology and Management Applied to Tissue Regeneration, UNIFESP. It was approved by the Research Ethics Committee (CEP), at Plataforma Brasil under number CAAE-64910117.8.0000.5505.

For the elaboration of the tool, the Design Thinking (DT) method was used Ferreira *et al.* ^12^ applied in four phases: Discovery, Definition, Development and Delivery.

###  Desk search 

 During the Desk search, a literature review was carried out to sort content, adapt the material, and prepare the tool. Articles from the databases and search sites, national and international IP books, national and international legislation, case law, and the WIPO Statistical Bank were reviewed, as well as the INPI, United States Patents and Trademark Office (USPTO), European Patents Office (EPO) and its booklets, to answer three questions:

1 - What is the interviewee’s problem?
2 - What does the interviewee use today to try to solve their problem?
3 - What can be done to improve the interviewee’s problem?

The following descriptors were used: patents, intellectual property, management of science, technology and innovation in health, surgical equipment, surgical instruments, dental equipment, dental instruments. The search was in the databases: SciELO, Pubmed, LILACS and in the search sites: Google and Google Scholar.

Research was also carried out based on the following keywords: innovative patents, patents on hospital medical and dental equipment, innovation in hospital medical and dental equipment; and medical, hospital and dental equipment. The research was made in the databases: SciELO, Pubmed, LILACS and in the search sites: Google and Google Scholar.

To find out what are the main questions and difficulties, in the EMHO patent filing process, EMHO inventors were interviewed. The inventors were selected from the list of students of the Master’s Degree Program in Science, Technology and Management Applied to Tissue Regeneration, following the inclusion criteria: students enrolled in 2015 and 2016, whose master’s project was based on the invention of EMHO and with approval by the CEP. Ten inventors were identified in these criteria at the research stage.

Individual interviews were scheduled with selected students, according to the criteria above. Prior to the interviews, they signed terms of free and informed consent, so the interviewees were aware of the content of the questions and agreed to participate. The interviews took place via videoconference and/or in person, with an average duration of 30 minutes each.

During the interview, which was conducted in an informal, directed conversation manner, the open questions, based on the Design Thinking (DT) methodology^13^ and on the booklets from INPI^14^:

1) What do you understand by patent?
2) In your opinion, what types of patents are there?
3) In your opinion, what is a patent for?
4) In your opinion, what can be patented and what cannot?
(5) In your view, what is the duration of a patent?
6) In your view, what is royalty?
7) In your opinion, how does the distribution of royalties work, especially when there are private companies and/or universities connected to the process?
8) When you invented the EMHO, did you think about filing a patent? Where did you look for information on how to proceed?
9) Have you started your patent filing process? If so, how did you do it?
10) Have you participated in any administrative procedures? Which ones?
11) What difficulties did you encounter in initiating the administrative patent procedure? Was there lack of information? What information? Were there any administrative barriers? Which ones? Were there very high administrative costs? Others?
12) How did the distribution of royalties of your patent occur? Did you sign a contract?
13) What are some of your major questions about patents?
14) Is there any other patent-related topic you would like to mention?

Through questions one to seven, the interviewees’ level of knowledge on “patents” was analyzed; the booklet from INPI^14^ was used to check the discursive answers, based on (Knows / Does not Know). Through questions 8 to 14, the interviewees’ questions and difficulties related to the patent registration process were analyzed.

After interviewing the selected students, a discussion group was created on WhatsApp, so that they could send their questions related to EMHO patent filing process to the group administrator (in this case, the designer of this research), whenever they had questions, for the following four months. The questions were answered by the group administrator and also recorded in a separate list. Then, along with the answers, the doubts and difficulties encountered by the interviewees in the EMHO patent filing process were used as an analysis tool.

After the “discovery” phase, the main problems and questions the interviewees had were defined. The answers to each question of the interview were compared, and then transferred to an Excel spreadsheet for easy visualization. Answer patterns (repeated themes) were grouped and listed, according to the frequency of responses. The themes represent topics of doubts and difficulties encountered by the interviewees. The questions sent to the WhatsApp group were also recorded in an Excel spreadsheet. Answer patterns (repeated themes) were searched, grouped, and listed, according to the frequency of occurrence. The two lists of themes and respective frequencies were put together and the percentages of respondents who mentioned each topic in their answers or doubts were calculated.

###  Development 

 A face-to-face brainstorming session was held with the interviewed inventors. The unified list of themes and the percentages of respondents, who mentioned the theme as doubt or difficulty, were presented to the interviewed inventors. The brainstorming was used to develop ideas of tools, answer questions and communicate solutions to the difficulties presented by the interviewees, and the best idea for a tool was chosen through voting and consensus.

In this phase, the chosen tool was elaborated in a power point (ppt) presentation as well as its content framework. An interactive video with guidelines was also made using PowToon (a British site with interactive videos to assemble content with several subscription plans, the prototype was made in the free version). The themes (doubts and difficulties) of the tool, raised in the previous step, were placed in a question and answer format and filled in based on the Desk search.

The prototype was presented to the inventors and to three users who volunteered (a prosecutor, a journalist and a user without university education) in order to evaluate and verify if the instrument met their needs. To do so, the following questions were asked:

1) How easy or difficult is it to use the tool? (0 - very difficult, 5 - very easy).
2) Does the content of the tool answer your questions about the subject? (0 - it does not answer, 5 - it answers).

The suggestions and comments obtained in the validation process were inserted in the prototype, so that the inventors could evaluate them again. This process can happen several times until the prototype reaches its final version. After the interviewees’ approval, the tool with complete content was designed.

The final version of the tool was published, and on the website of the Master’s Degree Program in Science, Technology and Management Applied to Tissue Regeneration, UNIFESP, for the general public to have access to it.

## Results

 From the Desk Research in the databases, papers were found for this research, which reported on patent issues, EMHO, innovations and invention descriptions. In search sites, booklets were found, as well as sites and papers on patents and EMHO.

The keywords “innovative patents” and “hospital medical and dental equipment” were the basis for this research.

In this DT phase, the answers found, to try to solve the problem, were:

1) The interviewee’s problem is the lack of information, from the moment they had the idea, because a connection between the subject patents and EMHO is missing.
2) The current options of information tools that the interviewees have and use are mainly the booklets from official bodies, institutions and personal websites.
3) The best that can be done is to connect the information on patents and EMHO, considering the moment when the interviewee had the idea.

In the interviews with the EMHO inventors, questions one to six were designed to know the audience and elucidate the researcher. Questions seven to fourteen were related to the events that occurred from the moment of the idea to the act of administrative filing.

Regarding the discussion group, no questions were asked in the group.

In the “definition” phase, to identify the doubts and difficulties, the answers were transferred to an Excel worksheet for easy visualization. Patterns of repeated responses (themes) were sought.

**Figure 1:**
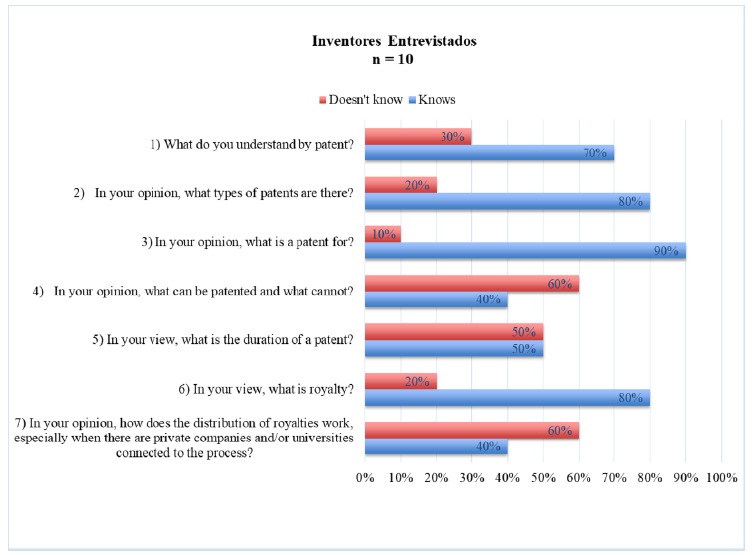
Answers to questions one to seven on patents, at a relative frequency.

From the questions that elucidate the interviewees’ knowledge (1-7), the open answers were verified, based on (Knows / Does not Know) (Fig. 1).

Questions 8-14 were about doubts and difficulties the interviewees had when having the idea and going through the administrative procedure to file a patent. 50% of the interviewees reported that they searched for information on how to proceed with invention and knowledge acquired during their Master’s Degree program (ICM); 40% invention and knowledge acquired before the Master’s Degree program (ICA) and 10% previous invention and knowledge acquired before the Master’s Degree program (IACM).

It is possible to observe that, ICM and IACM, knowledge on how to proceed to file a patent, was acquired during the Master’s Degree Program (total of 60% of the interviewees). 

In the last two questions, one respondent gave two answers, therefore its percentage surpassed 100%.

Five interviewees (50%) mentioned the lack of information when referring to this subject, and, along with the interviewees who mentioned lack of information and costs as barriers, the total sum was eight (80%).

Questions 11, 13, and 14 were the ones that best defined the content of the tool with the main topics; the others were regarded as subtopics.

Eight interviewees participated in the voting; five asked for a YouTube channel, two asked for applications and one, an e-book. The suggestion of an interactive website with interactive content and videos was accepted as the best idea to have technical content while having dynamic videos with guidelines.

The opportunity was taken to discuss a name for the tool, and, after seven votes, the following name was chosen: “*Faça Fácil Patentes Equipamentos Médicos*” (“Make Medical Equipment Patents Easy”).

The prototype was developed with content framework and an interactive video with guidelines.

The prototype was presented to the interviewed inventors, via WhatsApp and, for the volunteers, to evaluate and verify if the instrument met their needs. Questions were presented and, eight interviewees qualified it as very easy / it answers. The prototype was approved and the final version was developed.

Of the themes they brought up, eight (80%) of them were identified as lack of information; seven (70%) of the interviewees asked for a step-by-step on patents, from the idea to the administrative procedure of filing it; four (40%) asked for a step-by-step on search for precedents, and three (30%), private companies and partners.

The initial part of the tool voted during the brainstorming process was developed with topics and subtopics in the Word software. The themes were subdivided to know more about the step-by-step, the patent until the administrative filing procedure:

###  Learn more about patents 

 This item is to know about patents, in general, with interactive videos, a link with NIT/UNIFESP, as well as available forms.

- What is a patent?
- And the Invention, what is it?
- And the difference between Inventor and Patent Holder?
- The Brazilian Law and types of patents.
- What about the patent grant requirements?
- What cannot be patented?
- Second use patent?
- Innovation X Invention?
- What is the patent document like?
- Royalties?
- When is the university involved?
- What if you want to file a patent in other countries?
- What if you patent and do not use it?
- What cause breaches to your patent?
- Guidelines - interactive videos.
- Secrecy and confidentiality terms with companies and individuals.
- Useful links.

###  Step by step to create an innovative invention 

 This item was developed thinking about the individual who had the idea and needs to know how to proceed; the step-by-step from the idea, including the preparation of the administrative filing with INPI, as well as its technical guidelines, in drafting the application for filing the patent. It was made in an interactive format, aiming at an easier understanding.

1 - Invention.
2 - Document.
3 - Commercial potential.
4 - Is it patentable?
5 - What if the invention is not patent, what should I do with it?
6 - Patent filing?
7 - Search for precedents.
8 - Invention description based on INPI rules.
9 - Claims based on INPI rules.
10 - Design based on INPI rules.
11 - Designs based on INPI rules.
12 - Documents required for filing with INPI.
13 - Administrative Patent Procedure in INPI.
14 - Example of national charter - INPI.
15 - Fees.
16 - First examination for patent application.
17 - Guidelines.

###  Search for precedence (Step by Step) 

 This item describes how to make the search for precedence, presented by the titles:

- How to search?
- Guidelines: $, AND, OR, XOR, ANDNOT
- Free search sites

###  Medical equipment - EMHO 

 Information on EMHO was presented with its peculiarities, links to the governmental entities that finance these projects, as well as ANVISA and ABIMO (Brazilian Industry Association and papers about EMHO).

- Concept.
- Financing through BNDS, MCT, CNPq, FINEP, UNESCO (Links) that promote research through bids.
- ANVISA Rules (manual link).
- Patentability of medical methods.
- Guidelines: Copyright.

###  Patent examples 

 Each of these items/sub items has been filed with Desk search and with interactive videos created on PowToon.

From this company PowToon, an annual basic package was purchased, with which the researcher made the videos.

A professional was hired to create the art of the website with Wordpress CMS, to which most devices will have access (tables, phones, laptops and platforms MAC, WINDOWS, LINUX, ANDROID, OS X). 

**Figure 2:**
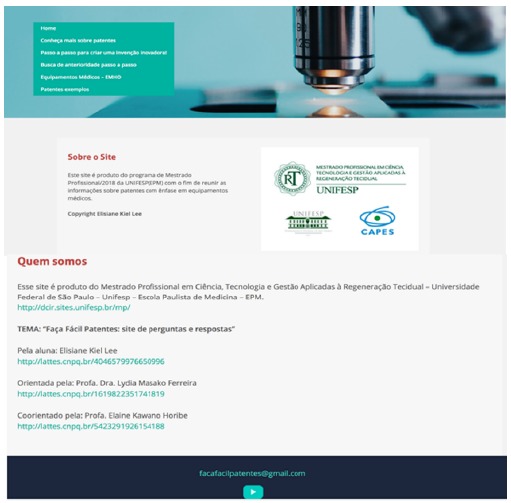
Main page layout.

The website layout can be visualized in the Fig. 2.

It was also necessary to subscribe to the registration of domain and hosting of the site with the company Locaweb.

The chosen registration was: www.facafacilpatentes.com.br

The site was validated and approved by the interviewees and volunteers.

After the official launching, the final version of the tool was published (link to the website www.facafacilpatentes.com.br), and link on the website of the Master’s Degree Program in Science, Technology and Management Applied to Tissue Regeneration of UNIFESP for the public in general to access it.

## Discussion

 The innovation system in the Brazilian health industry, especially regarding EMHO, is immature, because there is no connection between scientific and technological productions and patents^15^. There is no effective interaction among universities, companies and governmental bodies, even though it is fundamental to invest in R&D^16^.

Innovation has a breakthrough in medical technology, especially when it involves other industries such as bioengineering, materials engineering, molecular biology and innovation system, but the interaction among universities and companies in this area will certainly be an effective gain. However, the use of patents will be the most effective means of protection for new products^16^.

The EMHO system is growing, and in 2010 ABIMO detected a national growth of 50% in the last four years of research, in which most companies are micro-companies. The US official IP office (USPTO) shows an exponential increase in this area since 2009.

As stated in the introduction, in 2014, WIPO released the ranking of the largest patent applicants, and Brazil ranked 10^th^, with a volume of deposits far shorter than other countries. In the area of hospital medical and dental (EMHO) equipment, Brazil is in 11^th^ place, with 1.4%.

Brazil is also developing slowly in the patent filing system, as well as in EMHO. One of the great problems, in the words of the former president of the INPI Jorge de Paula Costa D’Avila, is the lack of knowledge on intellectual property^10^, which based the need to elaborate this research.

For this study, first, the literature was surveyed. In this area, there is very little information about innovative patents in EMHO, which brings the need to deepen the studies as a motivating source of scientific recognition. There is a lot of information about the patent process, mostly in the legal field, little information about EMHO and, practically nothing about innovative patents in EMHO. The search for EMHO with descriptors (DeCS) brought many files about inventions, but no material describing the concepts, economy, regularization and procedures. We resorted to the use of keywords, because the descriptors did not contemplate the most updated terms.

Thus, in order to understand the real needs of users of this tool, in the case of the inventors of EMHO, the DT method^12^ was used, addressing the problematic, focusing on approaching the user and understanding the situation and difficulties of the administrative phase of patent filing. In the literature, there is no study on patents on EMHO using DT as methodology; therefore this is the first study that used this methodology for the creation of this tool.

This methodology is new and had great importance for the final product, since it was elaborated with the participation of the inventors of EMHO. A questionnaire was applied^13^, with which we tried to check and answer some questions, not yet very clear for most readers.

In the questionnaire, the vast majority of the interviewees only acquired knowledge of the patent protection, in the master’s degree program, and even so, there was confusion and insecurity. In question eight, this attitude is shown. Of the interviewees, four (40%) had the idea and knowledge about IP protection in patents, prior to the master’s degree; five (50%), obtained previously to the knowledge acquired in the master’s degree and one (10%), obtained them in the master’s degree. Considering the knowledge acquired in the master’s degree, the total sum represents six (60%) of the interviewees. All of them have at least one specialization, that is, knowledge on IP was acquired late; moreover, this knowledge is still confused. For example, in question 12, about respondents having signed royalty share contracts, they stated that they had not signed a contract and that the Technological Innovation Center (NIT) only reported the format of the share, which is 30% for the inventors. Checking with the NIT on royalty agreements, there was an information disorder, since this document that they signed is the share agreement, for future royalties and in which the percentage of share among the inventors is emphasized, even though there is no third-party company interested in the patent.

Through the interviews and in the course of the contact with the inventors, it is possible to observe that NIT often confuses the inventors with information, perhaps due to a lack of preparation to pass on the information, or because they do not understand the inventor’s situation.

In Brazil, there are handbooks or booklets on patents, prepared by the National Institute of Intellectual Property - INPI and other national institutions; however, these publications do not contain specific guidelines related to EMHO, especially considering the moment when the inventor created the product. On the other hand, in the United States, there are yearly literary editions a, such as Medical devices patents by Sung^17^; however, this legislation does not apply to Brazil, despite some similarities.

Comparing with the existing information tools, INPI^14^, WIPO^18^, EPO^19^, booklets mostly focus on general patents. WIPO and EPO bring some regulations on medical devices, but the determinant here is the Brazilian legislation, which should be used as aid. In Brazil, regarding medical equipment, only papers related to the market prognosis were found, along with some characteristics and relations with patents and the like, but a lot of information is disperse.

The INPI Handbook^14^ presents an idea about what a patent and its peculiarities is, mainly in the administrative process, but the interviewees’ major question was related to when they had the idea: what should they do? Where should they go? And, mainly, how to have a more accessible language. In the INPI website, there is basic information to prepare a patent application, which was used as an aid to build the tool, especially for those who want to make the application for administrative deposit, since there is a whole formality to follow.

There are several sites available that briefly describe patents in general, explaining their use, the differences between patents and utility model, patent requirements, what cannot be patented and a very brief step-by-step. The difference of this elaborated tool is exactly the compilation of the information with emphasis in EMHO, guiding from the idea and, not only, from the administrative patent filing, which was one of the problems identified by the interviewees: the lack of information at the moment they had the idea, in addition to finding a lot of material with legal information is distant from the inventors. A single screen was made for the step-by-step, from the idea to be patented, including the administrative filing, for those who want to do it individually; another screen gives general patent information; another one presents the step-by-step search for precedence; another one contains information about EMHO and, lastly, a screen shows examples of successful patents.

There were barriers during the application of the DT methodology, which makes it easier to understand the user’s problem^12^. In the Development phase, in the brainstorming meeting to develop ideas of tools, it was very difficult to gather the ten interviewees, because all of them work in the health area, mostly as doctors and, after many failed attempts, this stage was accomplished by voting on paper, where everyone saw each other’s comments, and the best idea for a tool would be chosen through voting and consensus. At that time, the percentage of themes such as doubt or difficulty was also presented.

The number of interviewees (10) was the result of the selection of master’s degree program students who were filing patents which had to be approved with CEP and initiated before the NIT. They should be at a stage of the administrative procedure of patent filing before the NIT that really collaborated with the research.

This tool was developed to be an aid in format of questions and answers on patents with emphasis in medical equipment (EMHO), mainly, covering the mentioned difficulties and aiming at a closer reading to the user. The interviewees’ problems were identified, such as the lack of information and dynamism, so, in order to solve them and get to this tool, we tried to offer a simpler and more dynamic language, using the official bodies’ booklets (WIPO, EPO, USPTO, INPI) as a parameter.

After the official launch of the site, there will be online ads such as Google Adwords so the site may gain recognition and access, making it more and more relevant in the Google system when someone searches for patents in EMHO.

Smith and Sfekas^20^ report the importance for medical professionals to create innovation in products of the area and, consequently, make their patents. Therefore, the perspective of this product is to generate greater interactivity with the target audience. Through user profile surveys, it also aims at understanding the problems for future updates on the website and generating consultancies in intellectual property. Lastly, with a link to UNIFESP, it will be able to create incubators of startups in this industry. The impact is to spread information, in the health industry, reaching professionals, trying to solve problems, and being an incentive for the development of more patentable products. 

## Conclusion

 With this work, it was possible to develop a question and answer tool in patents in EMHO.
